# Immune-based Therapy Clinical Trials in Hepatocellular Carcinoma

**DOI:** 10.4172/2155-9899.1000376

**Published:** 2015-12-10

**Authors:** Dai Liu, Kevin F. Staveley-O’Carroll, Guangfu Li

**Affiliations:** 1 Department of Surgery, Medical University of South Carolina, Charleston, SC 29425, USA; 2 Hollings Cancer Center, Medical University of South Carolina, Charleston, SC 29425, USA; 3 Department of Surgery, University of Missouri, Columbia, MO 65212, USA; 4 Department of Molecular Microbiology and Immunology, University of Missouri, Columbia, MO 65212, USA; 5 Ellis Fischel Cancer Center, University of Missouri, Columbia, MO 65212, USA

**Keywords:** Hepatocellular carcinoma (HCC), Clinical trial, Immunotherapy, Adoptive immunotherapy, Vaccination, Immune checkpoint, Chemoimmunotherapy

## Abstract

Hepatocellular carcinoma (HCC) is the second leading cause of cancer-related mortality and continues to increase. Current standard of care for patients with HCC only provides limited therapeutic benefit. Development of innovative strategies is urgently needed. Experience with immunotherapy in HCC is quite early, but rapidly rise in the recent 15 years. Multifaceted immune-based approaches have shown efficacy in achieving disease regression, representing the most promising new treatment approach. Here, we classify the ongoing or completed clinical trials in HCC in terms of the immune strategies to be used and assess their clinical outcomes. The generated information may be helpful in the design of future immune-based therapies for achieving ideal tumor control and maximizing anti-tumor immunity.

## Introduction

Worldwide, HCC is the second leading cause of cancer-related mortality and continue to increase. Last 20 years, HCC has increased 62% to over 750,000 new cases annually [1,2]. In the united states, HCC is the fastest growing cause of cancer-related death and over 35,000 new cases are annually identified now [3]. Current treatments for HCC only provide limited benefit as survival is poor even for patients with local disease. HCC is refractory to classic chemotherapy and unsuitable for radiation treatment due to liver toxicity [4]. Surgical resection or ablation offers a small chance for cure. Liver transplantation is an effective treatment for cirrhosis and early tumors, but most patients are ineligible because recurrence is common and organs are scarce [5]. The receptor tyrosine kinase inhibitor (RTKI), sorafenib, was the first and only drug approved by the Food and Drug Administration (FDA) to treat unresectable HCC in 2008; however sorafenib only increases the median overall survival of patients from 7.9 to 10.7 months [6]. This small but statistically-significant therapeutic effect highlights the challenge in treating this devastating disease.

It is clear that even after cancer develops, the power of the immune system can be harnessed to suppress tumor growth [7–9]. Although experience with immunotherapy is quite early, multifaceted approaches have shown efficacy in achieving disease regression and even cure [10]. Manipulation of the immune system toward the rejection of established cancers as part of the standard of care is becoming closer to reality [11–13]. Studies of immune checkpoints in tumor-induced immune tolerance greatly advance immunotherapeutic drug development [14, 15]. The monoclonal antibodies against cytotoxic T-lymphocyte antigen 4 (CTLA-4) and programmed cell death protein 1 (PD-1) were respectively approved by FDA in 2011 and 2014 for the treatment of patients with advanced melanoma [16]. In March of this year, anti-PD-1 antibodies as the first immunotherapeutic agent for the treatment of squamous non-small cell lung cancer were approved by the US FDA [17]. These exciting progresses support the translation of immunotherapies to other cancers including HCC [18]. Searching for the term “cancer immunotherapy” at https://clinicaltrials.gov/ yields 1167 clinical studies, 124 of which are in phase III clinical trials, 669 of which are in phase II clinical trials and 575 of which are in phase I clinical trials. Among them, 27 clinical trials are used to treat patients with HCC. Here, we classify these ongoing or completed immunotherapy clinical trials and evaluate their therapeutic efficacy. The generated information may be helpful to maximize anti-tumor immunity and design future immune-based therapies for achieving ideal tumor control.

## Rapid rise of immunotherapy clinical trials in HCCs in recent 15 years

**Figure 1:**
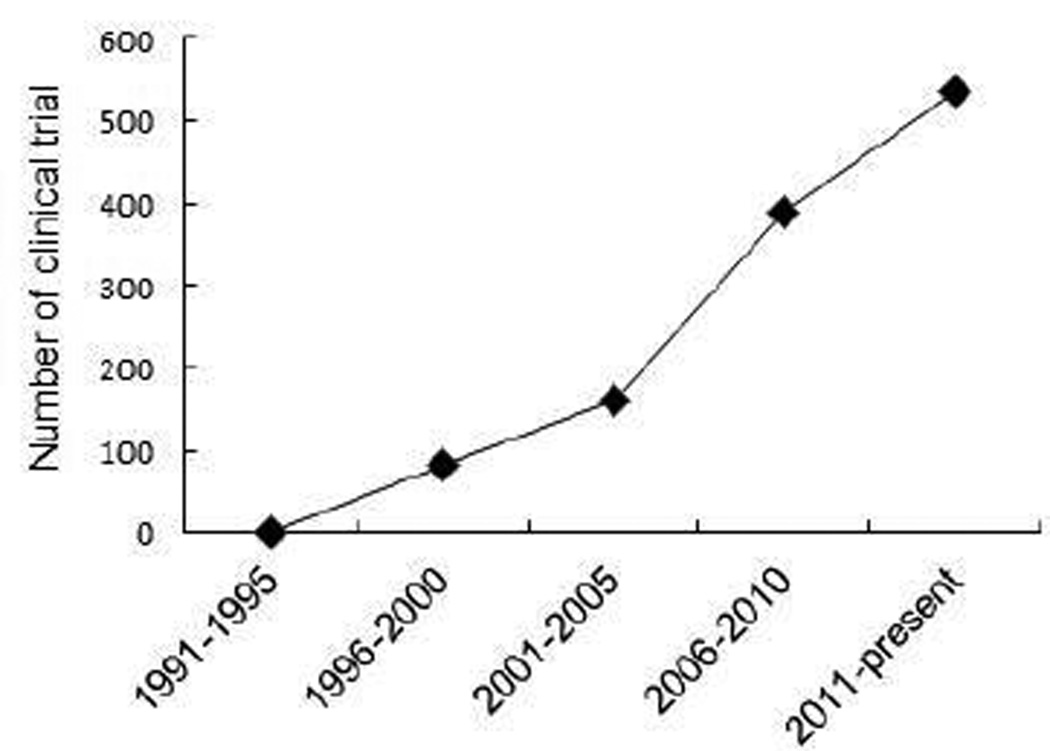
Cancer immunotherapy.

**Figure 2:**
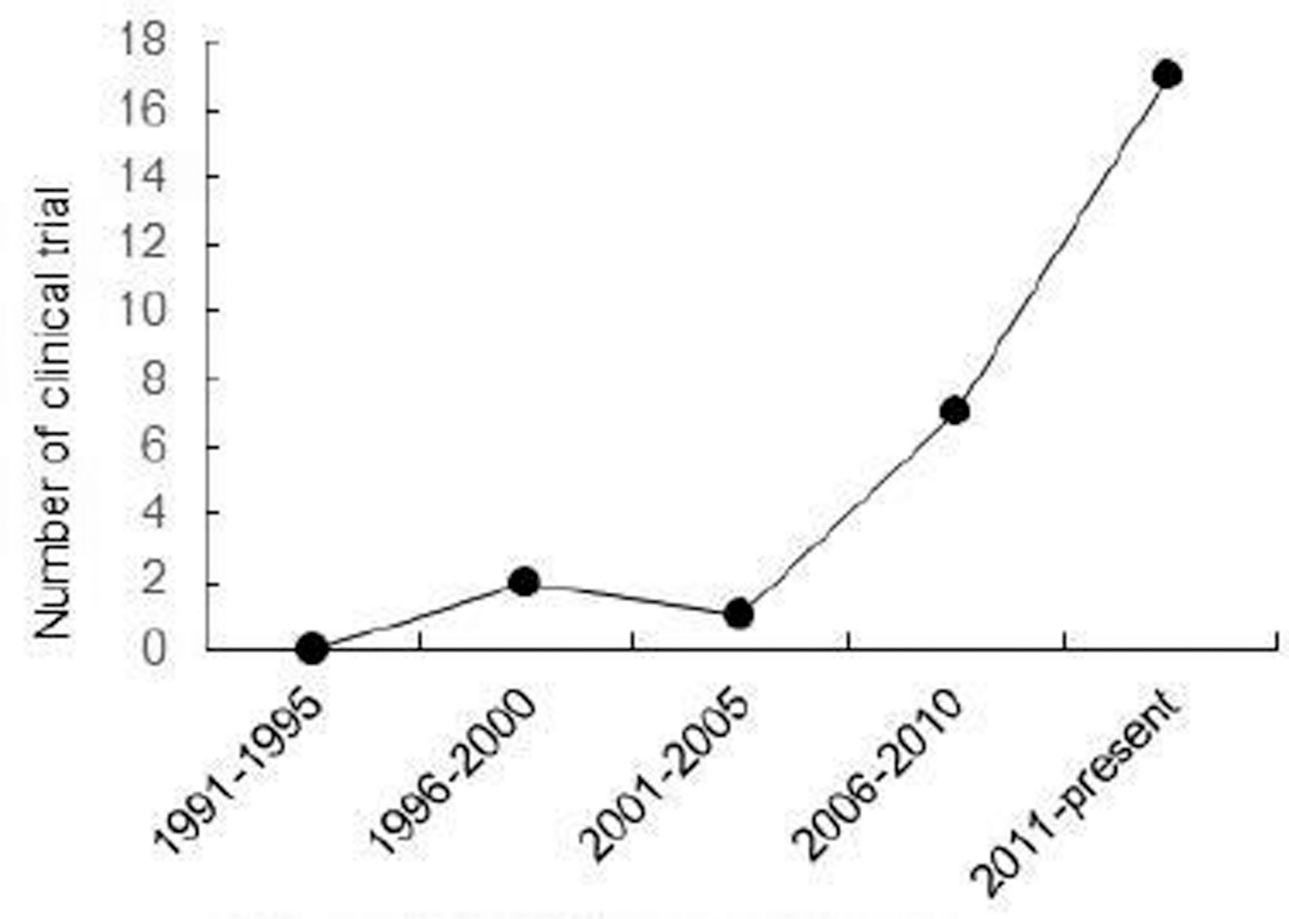
HCC immunotherapy.

While the function of immunity against cancers was recognized several decades ago, cancer immunotherapy from bench to bedside takes a long time, but rise rapidly in the recent 15 years. In late 1980s, French researchers discovered new protein receptors on the surface of T cells known as CTLA-4 [19]. James Allison, working now at the University of Texas MD Anderson Cancer Center in Houston, found that CTLA-4 functions as a brake to prevent T cells from generating powerful immune attacks. Initial functional studies suggested that antibodies-mediated blockade of CTLA-4 synergizes anti-CD28 antibodies to enhance T cell activation [20]. In 1996, Alison published a paper in Science showing that antibodies-mediated blockade of CTLA-4 destroyed tumors in mice [21]. In 2010, Bristol-Myers Squibb reported that anti-CTLA-4 antibodies treatment increased average lifespan of patients with metastatic melanoma from 6 months to 10 months [22]. Given this breakthrough success, the anti-CTLA-4 mAb was approved by FDA in 2011 for the treatment of patients with advanced melanoma [16]. Currently, basic and clinical scientists worldwide are working relentlessly to extend the promise to other cancers including HCC. Immune-based therapy clinical trials rise rapidly in recent 15 years. From 1991 to present, total 1167 immunotherapy clinical trials have been found in http://clinicaltrials.gov with about 90% of them conducted in recent 15 years (Figure 1). 27 of them are applied for the treatment of HCC with 25 conducted in the past 15 years (Figure 2). As an outstanding achievement, *Science* magazine named cancer immunotherapy as the biggest breakthrough of the year in 2013.

## Categories of immunotherapy in terms of strategies to be used

Opposed to traditional cancer treatments targeting tumors, cancer immunotherapy targets cancer patients’ immune system to fight off cancer cells. Based on strategies used in human HCC clinical trials, immunotherapies fall into four major categories: 1) adoptive immunotherapy, 2) therapeutic vaccination, 3) blockade of immune checkpoint, 4) combinational chemoimmunotherapy. To date, 27 immunotherapies have been conducted in human HCC including 13 in open studies with or without recruiting patients (Table 1) and 14 in closed studies with different status seen in Table 2.

## Adoptive immunotherapy

Adoptive immunotherapy is a form of passive immunization in which autologous effector cells are *ex vivo* sensitized and then given back to the cancer patients. The sensitized effector cells possess cytotoxic function to destroy cancer cells after adoptively transferring into cancer patients. The pioneer trial was conducted by Dr. Steven Rosenberg over 30 years ago with *ex vivo* activated cancer-fighting white blood cells [23]. As one of main immunotherapeutic strategies, adoptive immunotherapy is widely used in the current cancer clinical trials. About half of immunotherapy clinical trials in HCC (12 trials) are adoptive immunotherapy. In these trials, four types of immune cells are used in the adoptive immunotherapy, such as mixed killer cells (13), nature killer (NK) cells (2), nature killer T (NKT) cells (1) (Table 3), and chimeric antigen receptor (CAR) T cells.

## Cytokine-induced Killer(CIK) Cells

CIK cells are generated by *ex vivo* incubation of human peripheral blood mononuclear cells (PBMCs) or cord blood mononuclear cells with interferon-gamma (IFN-γ), anti-CD3 antibody, recombinant human interleukin 1 (IL-1) and recombinant human IL-2 [24–26]. The *ex-vivo* expanded CIK cells express CD3 and CD56, featuring a mixed T cell-like and NK cell-like phenotype [27]. The studies demonstrated CIK cells have potent, non-MHC restricted cytotoxicity against tumor cells [24, 25,28,29]. The high proliferation rate [25, 26], low risk of graft-vs-host disease [30] and easy availability contribute to their advantageous profile, making CIK cells a preferential adoptive immunotherapeutic approach for cancer patients [25, 31–33].

In 2000, the impact of a CIK therapy on the postsurgical recurrence rates was conducted in 72 HCC patients who had all undergone hepatic resection (NCT00699816) [34]. The median time for follow-up was 4.4 years. The recurrence rate in CIK cell treatment group was significantly lower (59%, 45 patients) than patients in the control group (77%, 57 patients). Also, the time to first recurrence was significantly longer in the CIK treatment group.

In 2004, the phenotypes of CIK effector cells, peripheral T lymphocyte subsets and dendritic cell (DC) subsets were investigated in 13 HCC patients who had liver cirrhosis and more than twenty years of chronic HBV infection [35]. 108 days after CIK cell infusion, the composition of lymphocyte subpopulations was still similar to the levels determined ten days after therapy. This indicates the long-term durable characteristics of CIK cells. Also, in another trial, CIK therapy reduced HBV burden from 1.85 × 10^6^ to 1.41 × 10^5^ copies of DNA/mL three months after therapy [36]. These results suggest that CIK cells are able to restrict viral infection in addition to tumor control.

In 2008, the impact of CIK therapy on tumor recurrence was conducted in 85 HCC patients who had received transcatheter arterial chemoembolization (TACE) and radio frequency ablation (RFA) [37]. After CIK cell infusions, the frequency of CD4^+^, CD3^+^, CD56^+^, and CD3^+^CD56^+^ T cell and the CD4^+^/CD8^+^ ratio were significantly increased (P<0.05); whereas the percentage of CD8^+^ cells decreased from 31.1 ± 7.8% to 28.6 ± 8.3% (P<0.05). The 1-year and 18-month recurrence rates of the study group were 8.9% and 15.6%, compared with 30.0% and 40.0% of the control group (both P value <0.05). Similar results were observed in a study performed by another group in 2010 [38]. The data suggest that CIK cell transfusion capably reduces the recurrence rate of HCC.

In 2009, a randomized study was conducted to investigate the outcome of postoperative CIK cells therapy in 127 HCC patients who underwent radical hepatic resection [39]. The results of a long follow-up demonstrated that adoptive CIK cell therapy can prevent or at least delay recurrence of HCC after hepatic resection. However, adjuvant CIK cell therapy does not seem to be able to improve the overall survival (OS).

In 2013, a retrospective study was conducted in 174 HCC patients from January 1999 to April 2012. Among them, 85 patients were given CIK cell infusion after treatment with TACE and RFA alone [40]. The results demonstrated that CIK cell infusion significantly prolonged the median survival time (MST) and the median progression-free survival (PFS) in patients compared to TACE or RFA monotherapy (MST: 56 months versus 31 months, P=0.023; PFS: 17 months versus 10 months, P<0.001). The 3-, 5-, and 10-year OS was also significantly higher in the CIK group (P ≤ 0.005). This result was supported by another nonrandomized controlled clinical trial conducted in 146 patients [41].

In addition, two groups reported that infusion of *ex vivo* activated tumor infiltrating lymphocytes (TILs) also decreased the cancer recurrence and prolonged the tumor-free time compared to the control group without receiving TILs [34, 42].

In summary, CIK cell infusion in combination with other standard of care is an effective therapy which significantly delays recurrence and increases survival of patients with HCC.

## NK cells

NK cell is a type of cytotoxic lymphocyte critical to the innate immune system. Phenotypically, NK cells are defined as CD56^+^ CD3^−^ in humans. Receptor diversity allows NK cells exert the different function in response to the challenge with different pathogens including virus-infected cells and neoplastic cells [43, 44]. The number of NK cell in the peripheral and tumor is positively correlated with the survival and prognosis of HCC patients [45]. However, NK cells were functionally impaired in advanced HCC patients [46, 47]. Impaired NK cells were found to be associated with increase of regulatory T cells (Tregs) [48] and myeloid-derived suppressor cells (MDSCs) [49], resulting in reduction of anti-tumor immune response. Treatment of NK cells with IL-2, IL-12 and IFN-α/β is able to activate their cytotoxic capacity [43, 44]. Activated NK cells release cytokines and chemokines to improve both innate and adaptive immune response [50].

A group in the University of Miami has characterized NK cells extracted from living donor liver graft [51]. They observed that the activated NK cells with IL-2 and IFN-γ generate strong cytotoxicity [51]. A phase I safety study of liver NK cell therapy for hepatoma liver transplantation (NCT01147380) was started in July, 2010 and completed in December, 2014. An ongoing phase II clinical trial (NCT02008929) was initiated in August, 2014. This trial evaluates the safety and efficacy of MG4101 (*ex vivo* expanded allogeneic NK cells) as a secondary treatment after curative liver resection on advanced HCC patients with a high risk of recurrence. Both studies have not published the results.

## NKT cell

NKT cells refer to a heterogeneous group of CD1d-restricted T cells that have phenotypic and functional characteristics of both T cells and NK cells, evidenced by coexpressing a heavily biased, semi-invariant T-cell receptor and NK cell markers CD56, CD161 [52, 53]. NKT cells can regulate diverse immune responses and produce large quantities of cytokines following activation. Human NKT cells comprise a small population of innate T lymphocytes with 0.5% in healthy liver and 0.02% in blood [54, 55]; however, they are critical players in the regulation of anti-tumor immunity [56–59]. Subsets of NKT cells can play distinct and sometimes opposing roles [60]. In cancer, type I NKT cells, defined by their invariant TCR using Vα14Jα18 in mice and Vα24Jα18 in humans, are mostly protective. Type I NKT cells produce IFN-γ to activate NK and CD8^+^ T cells and stimulate DCs to produce IL-12. In contrast, type II NKT cells, characterized by more diverse TCRs recognizing lipids presented by CD1d, mainly inhibit tumor immunity [59]. Type I and type II NKT cells axis as a mechanism greatly influence other immune responses [59, 61,62]. Regulation of type I and type II NKT cells balance could be used as a strategy in designing cancer immunotherapies [63].

In January, 2013, a group in China started a phase I randomized controlled trial to investigate the efficacy and safety of autologous NKT cells infusion in advanced HCC patients (NCT01801852). They estimate to complete this study in 2017. A similar phase I clinical trial was proposed by another group in Arizona in 2009 (NCT00909558). The purpose is to assess the safety and effectiveness of NK cell and NKT cell-based autologous adoptive immunotherapy in subjects with different solid cancers including HCC, but this study has suspended participant recruitment without reason.

## Chimeric antigen receptor (CAR) T cells

CAR T cells are the genetic engineering of T cells through the introduction of a chimeric antigen receptor (CAR) [64]. The genetically modified T cells target tumors through the expression of a CAR. CAR design and elements required for the successful eradication of malignancies have been widely studied and tested in various cancers. The results suggest that CAR T cell therapy is a highly promising treatment for cancer and generates the favorable preclinical and clinical results [65]. In 2010, FDA approved a phase I/II study of CAR T cells in subjects with different cancers by targeting VEGFR2 (NCT01218867). HCC patients without hepatitis B and C are included; however, no result has been posted.

## Cancer vaccine

Cancer vaccines help the immune system to recognize and attack cancer cells. There are two types of cancer vaccine. Treatment of existing cancer is known as therapeutic cancer vaccines. Prevention of cancer from developing in healthy people is known as preventive vaccine. While prophylactic HBV and HCV vaccines contribute to the decrease of HCC patients [66], therapeutic vaccines for HCC are still awaited due to presence of other risk factors and the increased prevalence of non-alcoholic fatty liver disease [67, 68]. To date, eight vaccine clinical trials in HCC patients were completed or are ongoing with 4 trials in phase I, 2 in phase I/II and 1 in phase III (Table 4). The vaccines targeting HBV and HCV are beyond the scope of current review and not introduced here.

Butterfield et al. used CD8^+^ T-cell epitopes specific for alpha fetoprotein (AFP) to carry on the first HCC vaccine clinical trial. The results showed the generation of AFP-specific T-cell responses in vaccinated subjects [69]. Subsequently, Butterfield et al. conducted another phase I/II trial with autologous DCs *ex vivo* pulsed by AFP epitopes. These DCs are large, granular lymphocytes with high expression of MHC class I, MHC class II, and CD86 and expected to enhance immune response [70]; however, this trial only resulted in transient CD8^+^ T-cell responses, possibly caused by the lack of CD4^+^ help [71]. The similar clinical trial was conducted in 2010 by another group from France (NCT01128803), but was terminated without result reported. In addition, autologous DCs pulsed *ex vivo* with the lysate of the autologous tumor [72], HepG2 cells [73] and telomerase peptides [74], have been evaluated in human clinical trials. Unfortunately, all of the studies only showed limited improvements in clinical outcomes. A new phase I trial on DC vaccine was registered last year and is now recruiting participants (NCT01974661). Some strategies including DC immunotherapy combined with local radiation [75] or TACE [76] were also used in HCC clinical trials, but no significant impact on prevention of tumor recurrence was detected. In January, 2015, a phase III clinical trial was started to seek the therapeutic benefit of hepcortespenlisimu (V5) in subjects with advanced HCC (NCT02232490). Efficacy of this trial will be evaluated by measuring AFP level over the treatment and monitoring tumor change in initial time and end time by CT-scan.

In summary, current vaccine monotherapy doesn’t generate significant clinical outcome in patients with HCC.

## Blockade of checkpoint

Immune checkpoints are critical modulators in the immune system that either turn up a signal (co-stimulatory molecules) or turn down a signal (co-inhibitory molecules). The balance between costimulatory signals and inhibitory immune checkpoints determines the cytotoxic T-cell activation and intensity of immune response [77, 78]. It is now clear that tumors modulate immune checkpoints as one of the mechanisms to escape immune surveillance and rejection [79]. Since around 2010 checkpoint molecules have been increasingly considered as new targets for cancer immunotherapies due to the effectiveness of two checkpoint inhibitor drugs in the treatment of advanced melanoma [80]. Owing to the great achievement, immune checkpoint blockade therapy sheds light on other solid tumors including HCC. Antibodies-mediated blockades of CTLA-4 and PD-1 are currently being tested in HCC clinical trials. Five anti-PD-1 antibodies and three anti-PD-L1 antibodies are currently under development (Table 5), emphasizing the growing interest in these immune checkpoint pathways as a target for cancer therapy [77].

A phase II trial of humanized monoclonal antibody against CTLA-4 (tremelimumab) in HCC patients was started in 2009 and completed in 2012 (NCT01008358) [81]. In this trial, the ability of tremelimumab in triggering tumor responses was explored in HCV-infected patients with HCC and refractory to other therapies. Besides, the effect on the replication of the virus was also analyzed. The trial demonstrated that tremelimumab showed a safety profile and signs of antitumoral and antiviral effects that warrant further investigation in larger clinical trials [71]. In 2013, a phase I clinical trial of tremelimumab together with TACE, RFA, stereotactic body radiation therapy (SBRT) or cryoablation in subjects with HCC was started (NCT01853618). Patients with advanced liver cancer but refractory to other treatments were recruited. The safety and effectiveness of tremelimumab with TACE or RFA were tested. A phase I/II trial of anti-PD1 antibody (CT-011) in advanced HCC was initiated in 2009 but stopped due to slow accrual (NCT00966251). Another phase I dose escalation study of anti-PD-1 antibody (nivolumab) was initiated in 2012 and is currently ongoing (NCT01658878). This trial is to investigate the safety, immunoregulatory activity, pharmacokinetics, and preliminary antitumor activity of nivolumab in advanced HCC patients with or without chronic viral hepatitis. The initial findings were announced in the 51^st^ Annual Meeting of the American Society of Clinical Oncology (ASCO) in May, 2015 [82]. Investigators demonstrated that the estimated survival rate in evaluable patients (n=47) was 62% at 12 months. The durable partial responses and complete responses were detected in one out of five nivolumb-treated patients. The safety profile of nivolumab is generally consistent with that previously-reported in other tumor types. These encouraging preliminary data support the ongoing evaluation of nivolumab in this patient population.

## Chemoimmunotherapy

Chemoimmunotherapy is chemotherapy combined with immunotherapy. Chemotherapy uses different drugs to kill tumor cells or slow tumor growth; immunotherapy uses treatments to stimulate or restore the ability of the immune system to reject cancers. Accumulating data suggest that antitumor activity of some conventional chemotherapeutic drugs is, in part, associated with their ability to activate anti-tumor immune response [83]. Therefore, successful development of chemoimmunotherapeutic strategies, that both maximize tumor regression and the antitumor immune activity, are expected to achieve long-term clinical benefit in cancer control. Over the past 10 years, a great amount of preclinical studies in animal models validated this concept as combinational chemoimmunotherapy improved clinical outcome in different cancers. Using our clinically relevant murine model of HCC, we previously demonstrated that the combination of sunitinib (an FDA-approved chemotherapeutic drug for the treatment of ccRCC and GIST) with adoptive transfer of tumor antigen-specific CD8^+^ T cells leads to durable long term regression of established HCC tumors [84]. Given the exciting results gained in preclinical studies, chemoimmunotherapy has been introduced into HCC clinical trial (Table 6).

In 1999, a phase II study of doxorubicin and IL-2 in unresectable HCC was initiated and completed in 2001 without results reported. This trial is to evaluate the immunological response and tumor response in patients with unresectable HCC to doxorubicin and protracted recombinant IL-2. In addition, PFS and OS of this patient population after treatment with this regimen is assessed. Another two chemoimmunotherapies were initiated in 2012 and 2015, but not recruiting participants.

Prerequisite for successful chemoimmunotherapy requires chemotherapy-induces favorable environment allowing immunotherapy to exert effective cancer cytotoxic function. Ideal chemotherapy drug is capable to generate immunogenic cell death and block tumor-induced immune tolerance [85] which involve in the release of tumor antigens, emission of danger-associated molecular patterns (DAMP), the activated expression of the pattern recognition receptor (PRR) Toll-like receptor 3, rapid secretion of type I IFNs, and the release of the chemokine CXCL10, etc. In addition, the effect of chemotherapy on antitumor immunity is a drug-, dose-, and schedule-dependent manner [86]. Thus, to design effective chemoimmunotherapy regimens, clinical investigators should consider how chemotherapy impacts the immune system and perform the early-phase clinical studies for defining the optimal drug dose and timing in relation to immunotherapy [86].

## Future Perspectives

Notably, the powers of immune system can be exploited to destroy tumors. Given immune system’s amazing power with capacity for memory, exquisite specificity plus central and universal role in human biology, immunotherapy has the potential to achieve complete, long-lasting remissions and cancer cures, representing the most promising new cancer treatment approach with few or no side effects.

## Figures and Tables

**Table 1:** Open studies of immunotherapy clinical trials in HCC.

Intervention	Not yet recruiting	Recruiting	Unknown	Total
Adoptive therapy		6		6
Therapeutic vaccine		1	1	2
Blockade of checkpoint	1	2		3
Combinational chemoimmunotherapy	2			2
**Total**	3	9	1	13

**Table 2:** Closed studies of immunotherapy clinical trials in HCC.

Intervention	Completed	Terminated	Withdrawn	Suspend	Active Not recruiting	Total
Adoptive therapy	4	1		1		6
Therapeutic vaccine	2	1	2		1	6
Blockade of checkpoint						
Combinational chemoimmunotherapy	2					2
**Total**	8	2	2	1	1	14

**Table 3:** Clinical trials applying adoptive therapy for treatment of HCC.

Registered No.	Intervention	Start Year	Patient	Phase	Sponsor	Status
NCT01749865	CIK	2008	After radical resection	III	Sun Yat-sen University	Completed
NCT00562666	Gamma delta T lymphocytes	2008	Non operable tumor	I	Rennes University Hospital	Terminated
NCT00699816	Immuncell-LC: *in vitro* activated T cells	2008	Stage of I or II, tumor completely removed by resection	III	Green Cross Cell Corporation	Completed
NCT00769106	CIK	2008	After radical resection	III	Sun Yat-sen University	Completed
NCT01024530	IKCs: *ex vivo* expanded autologous IKCs plus TACE	2009	Never receive TACE treatment	II/III	Shin Kong Wu Ho-Su Memorial Hospital	unknown
NCT01147380	Liver NK cell inoculation with liver transplantation	2010	Liver transplant recipient with HCC	I	Seigo Nishida, University of Miami	Completed
NCT01174121	Short-term cultured, autologous TILs	2010	Metastatic HCC standard chemotherapy	II	National Cancer Institute (NCI)	Recruiting
NCT01462903	Autologous TILs plus IL-2	2011	Metastatic HCC	I	Sun Yat-sen University	Recruiting
NCT01758679	CIK and Licartin	2012	Postoperative HCC	IV	Tianjin Medical University Cancer Institute and Hospital	Recruiting
NCT01801852	Autologous NKT cell infusion	2013	Refractory to conventional treatment	I	Chinese PLA General Hospital	Recruiting
NCT02026362	CTL induced by DC loaded with multiple antigens	2013	After complete resection	I	SYZ Cell Therapy Co.	Recruiting
NCT01821482	DCs and CIKs	2013	After complete resection or TACE	II	Guangxi Medical University	Not yet Recruiting
NCT01897610	Immuncell-LC: activated T cells, with Nexavar	2013	Stage III and IV, Nexavar treated or ready to be treated	II	Green Cross Cell Corporation	Recruiting
NCT02008929	MG4101: *ex vivo* expanded allogeneic NK Cell	2014	After curative resection	II	Samsung Medical Center	Recruiting
NCT01914263	Cord Blood-derived CIKs	2014	After radical resection	I	Alliancells-PuRui Biocience Co., Ltd.	Recruiting
NCT02487017	DC-CIK combined with TACE	2015	After TACE treatment	II	Shenzhen Hornetcorn Biotechnology Company	Recruiting

HCC: Hepatocellular Carcinoma; CIK: Cytokine-induced Killer; IKC: Immune Killer Cell; CTL: Cytolytic T lymphocyte; DC: Dendritic Cell; IL: Interleukin; NK: Natural Killer; NKT: Natural Killer T; TACE: Transarterial Chemoembolization; TIL: Tumor Infiltrating Lymphocyte

**Table 4:** Clinical trials of tumor vaccine on hepatocellular carcinoma.

Registered No.	Intervention	Vaccine Type	Start Year	Patient	Phase	Sponsor	Status
NCT00610389	DC loaded with autologous tumor	Therapeutic vaccine	2008	Metastatic HCC	II	Clinica Universidad de Navarra	Unknown
NCT01128803	Autologous DCs loaded with AFP peptides	Therapeutic vaccine	2009	AFP ≥ 40 ng/ml	I/II	Nantes University Hospital	Terminated
NCT00669136	AFP + GM-CSF plasmid prime and AFP adenoviral vector boost	Therapeutic vaccine	2009	Locoregionally treated HCC	I/II	Lisa H. Butterfield, Ph.D.	Terminated due to poor accrual
NCT01828762	Autologous DCs incubated with irradiated autologous tumor stem cells and suspended in GM-CSF	Therapeutic vaccine	2012	Candidates for resection	I	Cellular Biomedicine Group Ltd.	Completed
NCT01522820	DEC-205/NY-ESO-1 fusion protein CDX-1401 vaccine	Therapeutic vaccine	2012	After resection and TACE	I	Roswell Park Cancer Institute	Not recruiting
NCT01974661	Allogenic DC based therapeutic vaccine	Therapeutic vaccine	2013	Not eligible for curative treatment or TACE	I	Immunicum AB	Recruiting
NCT01923233	ALLOSTIM(TM) in-situ vaccine in combination with RFA	Therapeutic vaccine	2013	Refractory HCC	I	Immunovative Therapies, Ltd.	withdrawn prior to enrollment
NCT02232490	hepcortespenlisimut-L (V5)	Therapeutic vaccine	2015	Advanced HCC	III	Lisichansk Regional Tuberculosis Dispensary	Recruiting

AFP: α-fetoprotein; DC: Dendritic Cell; GM-CSF: Granulocytes Macrophage Colony-Stimulating Factor; HCC: Hepatocellular Carcinoma; TACE: Transarterial Chemoembolization; RFA: Radiofrequency Ablation

**Table 5:** Clinical trials of checkpoints blockade on hepatocellular carcinoma.

Registered No.	Intervention	Patient	Phase	Start Year	Sponsor	Status
NCT01008358	CP 675,206 (tremelimumab) - Anti-CTLA antibody	Unresectable HCC	II	2008	Clinica Universidad de Navarra, Universidad de Navarra	Completed
NCT00966251	Pidilizumab – anti-PD1 antibody	Not operational HCC	I/II	2009	CureTech Ltd.	Terminated due to slow accrual
NCT01658878	Nivolumab – anti-PD1 antibody	Advanced HCC	I	2012	Bristol-Myers Squibb	Recruiting
NCT01853618	Tremelimumab – anti-CTLA4 antibody	Advanced HCC	I	2013	National Cancer Institute (NCI)	Recruiting
NCT02519348	MEDI4736 (anti-PD-L1 antibody), tremelimumab (anti-CTLA4 antibody)	unresectable HCC	I/II	2015	MedImmune LLC	Not yet recruiting

**Table 6:** Clinical trials of combination therapy on hepatocellular carcinoma.

Registered No.	Intervention	Start Year	Patient	Phase	Sponsor	Status
NCT00004248	Doxorubicin and IL-2	1999	Patients with liver cancer that cannot be removed by surgery	II	Roswell Park Cancer Institute	Completed
NCT01522820	Vaccine therapy with or without Sirolimus	2012	Patients with NY-ESO-1 expressing solid tumors	I	Roswell Park Cancer Institute, National Cancer Institute	Ongoing, but not recruiting
NCT02562755	Vaccinia virus-based immunotherapy + Sorafenib vs Sorafenib alone	2015	Advanced HCC without prior systemic therapy	III	SillaJen, Inc.	Not yet recruiting
